# Guidance for the design and analysis of cell-type-specific DNA methylation epidemiology studies

**DOI:** 10.1093/bib/bbaf427

**Published:** 2025-08-21

**Authors:** Emma M Walker, Emma L Dempster, Alice Franklin, Anthony Klokkaris, Barry Chioza, Jonathan P Davies, Georgina E T Blake, Joe Burrage, Stefania Policicchio, Rosemary A Bamford, Leonard C Schalkwyk, Jonathan Mill, Eilis Hannon

**Affiliations:** Department of Clinical and Biomedical Sciences, University of Exeter Medical School, University of Exeter, Barrack Road, Exeter, Devon, EX2 5DW, United Kingdom; Department of Clinical and Biomedical Sciences, University of Exeter Medical School, University of Exeter, Barrack Road, Exeter, Devon, EX2 5DW, United Kingdom; Department of Clinical and Biomedical Sciences, University of Exeter Medical School, University of Exeter, Barrack Road, Exeter, Devon, EX2 5DW, United Kingdom; Department of Clinical and Biomedical Sciences, University of Exeter Medical School, University of Exeter, Barrack Road, Exeter, Devon, EX2 5DW, United Kingdom; Department of Clinical and Biomedical Sciences, University of Exeter Medical School, University of Exeter, Barrack Road, Exeter, Devon, EX2 5DW, United Kingdom; Department of Clinical and Biomedical Sciences, University of Exeter Medical School, University of Exeter, Barrack Road, Exeter, Devon, EX2 5DW, United Kingdom; Department of Clinical and Biomedical Sciences, University of Exeter Medical School, University of Exeter, Barrack Road, Exeter, Devon, EX2 5DW, United Kingdom; Department of Clinical and Biomedical Sciences, University of Exeter Medical School, University of Exeter, Barrack Road, Exeter, Devon, EX2 5DW, United Kingdom; Italian Institute of Technology Center for Human Technologies (CHT), Via Enrico Melen, 83, 16152 Genova GE, Italy; Department of Clinical and Biomedical Sciences, University of Exeter Medical School, University of Exeter, Barrack Road, Exeter, Devon, EX2 5DW, United Kingdom; School of Life Sciences University of Essex, Wivenhoe Park, Colchester, Essex, CO4 3SQ, United Kingdom; Department of Clinical and Biomedical Sciences, University of Exeter Medical School, University of Exeter, Barrack Road, Exeter, Devon, EX2 5DW, United Kingdom; Department of Clinical and Biomedical Sciences, University of Exeter Medical School, University of Exeter, Barrack Road, Exeter, Devon, EX2 5DW, United Kingdom

**Keywords:** epigenetic, epidemiology, cell-specific, DNA methylation

## Abstract

Recent studies on the role of epigenetics in disease have focused on DNA methylation (DNAm) profiled in bulk tissues limiting the detection of the cell type affected by disease-related changes. Advances in isolating homogeneous populations of cells now make it possible to identify DNAm differences associated with disease in specific cell types. Critically, these datasets will require a bespoke analytical framework that can characterize whether the difference affects multiple or is specific to a particular cell type. We take advantage of a large set of DNAm profiles (*n* = 751) obtained from five different purified cell populations isolated from human prefrontal cortex samples and evaluate the effects on study design, data preprocessing, and statistical analysis for cell-specific studies, particularly for scenarios where multiple cell types are included. We describe novel quality control metrics that confirm successful isolation of purified cell populations, which when included in standard preprocessing pipelines provide confidence in the dataset. Our power calculations show substantial gains in detecting differentially methylated positions for some purified cell populations compared to bulk tissue analyses, countering concerns regarding the feasibility of generating large enough sample sizes for informative epidemiological studies. In a simulation study, we evaluated different regression models finding that this choice impacts on the robustness of the results. These findings informed our proposed two-stage framework for association analyses. Overall, our results provide guidance for cell-specific epigenome-wide association studies, establishing standards for study design and analysis, while showcasing the potential of cell-specific DNAm analyses to reveal links between epigenetic dysregulation and disease.

## Introduction

Recent years have seen increased attention to the role of genetics and gene regulation in development and complex disease [1, 2] facilitated by sequencing and array-based technologies. This includes studies of the epigenome, which encompasses a diverse number of chemical modifications to DNA and nucleosomal histone proteins that directly influence gene expression. In contrast to the genome, the epigenome is highly dynamic, varying across development, between cell types, and in response to the environment. Consequently, this means that careful consideration of study design is required when investigating relationships between epigenetic variation and complex traits [3, 4]. The most studied epigenetic modification in the context of human health and disease is DNA methylation (DNAm) [5, 6], which involves the addition of a methyl group to a cytosine. Most existing association studies leverage the high-throughput nature of microarrays to profile DNAm at hundreds of thousands of positions across the genome, meaning it is feasible both practically and financially to identify disease-associated variation for large sample numbers.

It is well-established that a DNAm profile is primarily defined by tissue or cell type [7–11]. Therefore, the choice of tissue for profiling influences both the analytical results obtained and the nature of conclusions that can be drawn from these. Profiling the primary affected tissue, for example, blood for immune-related traits and the prefrontal cortex (PFC) for neuropsychiatric and neurodegenerative diseases, is pertinent to making mechanistic inferences about how DNAm variation contributes to a particular trait. However, even these ‘bulk’ tissues represent a heterogeneous mix of cell types. Each of these cell types has its own DNAm profile, with the resulting profile of the bulk tissue being an aggregate of those from the constituent cell types. As the proportion of each cell type within a sample can vary across individuals, systematic differences in cellular proportions that correlate with the phenotype of interest may manifest as differences in the overall DNAm profile [12]. To minimize potential false positive associations, quantitative covariates that capture the cellular composition of each sample are typically included in statistical analyses [12]. The major caveat of analyzing DNAm in bulk tissue is that it does not enable the identification of which cell types are affected by the detected differences. In addition, subtle changes or differences in rarer cell types may be missed as they compete against the background signal from more abundant cell types. Elucidating which cell type(s) are affected by DNAm differences is critical for determining the genes and biological processes associated with specific complex traits and ultimately identifying novel targets for preventing and treating disease.

The case for generating cell-specific DNAm profiles to facilitate cell-specific analyses of epigenetic variation in disease is compelling. Although methods for single-cell DNAm profiling have been developed [13–15], these approaches are not currently amenable to large-scale analyses of human disease. Instead, techniques such as fluorescence activated nuclei sorting (FANS) and laser capture microdissection can be used to isolate purified cell populations from bulk tissue prior to genome-wide assays and have been applied to tissues such as whole blood [9, 16] and cortex [17–19]. Many datasets generated from these methods are small, aimed primarily at generating reference profiles for characterization of those cell types or as input for reference-based deconvolution algorithms that estimate the cellular composition from bulk tissue profiles [20]. There are, however, cases where these data have been used for epigenetic epidemiology to identify variation in DNAm associated with disease [19, 21–25].

Although the primary tissue for a particular disease may be obvious, the specific cell type involved is often less clear with cell-specific epigenome-wide association studies (EWAS) typically including multiple isolated cell types. In these scenarios, the objective differs from the traditional bulk tissue EWAS. Not only is the goal to identify loci in the genome where differences in DNAm correlate with the outcome of interest, but also to characterize whether the difference manifests across multiple cell types or is specific to a particular cell type. This will require a change in analytical approach because existing statistical approaches based on standard linear regression are potentially inadequate for analyzing these data. First, unlike most traditional bulk tissue analyses, multiple samples will be profiled for each individual. This threatens a major assumption of linear regression, that the observations are independent. Second, the statistical framework must capture differences between cases and controls that could be present in all cell types or are only present in a subset of cell types, by estimating case-control differences per cell type and assess whether these are statistically consistent.

In this manuscript, we evaluate the effects on study design, data preprocessing, and statistical analysis for cell-specific studies of DNAm, particularly where multiple cell types are considered, by taking advantage of a large DNAm dataset including five different purified cell populations isolated from PFC. First, we describe necessary extensions to established quality control pipelines that ensure that the isolation of purified cell populations has been successful. Second, we investigate the effect on statistical power of an association study that uses cell-specific DNAm data. Third, we assess the impact of how the data are normalized on the variance in DNAm. Finally, we evaluate multiple statistical frameworks for analyzing cell-specific DNAm data, using two simulation scenarios: a null association study and an association study with differentially methylation positions introduced. With these results, we provide guidance for the field in anticipation of future cell-specific EWAS with the objective of establishing standards for how these studies are designed, analyzed, and interpreted.

## Methods

### Isolation of neural nuclei from post-mortem brain tissue

Post-mortem tissue from 287 adult donors (aged 18–108 years old) was provided from multiple international brain banks (Cambridge, Edinburgh, Stanley, King’s College London, Harvard, UCLA, Oxford, Miami, Douglas Bell, Pittsburgh and Mount Sinai Brain Banks). Tissue was collected under approved ethical regulation at each centre and transferred through Materials Transfer Agreements. Post-mortem human PFC samples were processed using a FANS protocol developed by our group [26]. The gating strategies we implemented are shown in Supplementary Fig. S1.

### Methylomic profiling

DNA was extracted from frozen nuclei aliquots using a modified proteinase K-based extraction method developed specifically for these sample types [27]. 500 ng of genomic DNA from each sample was treated with sodium bisulfite using the Zymo EZ-96 DNA Methylation-Gold™ Kit (Cambridge Bioscience, UK) according to the manufacturer’s standard protocol. All samples were then processed using the EPIC 850K array (Illumina Inc, CA, USA) according to the manufacturer’s instructions, with minor amendments and quantified using an Illumina iScan System (Illumina, CA, USA). All sorted fractions from two randomly selected individuals were assigned to each BeadChip, within which the location of each fraction was randomized. Altogether, 293 Total (unsorted), 290 NeuNPos (NeuN+), 286 SOX10Pos (NeuN-/SOX10+), 27 IRF8Pos (NeuN-/SOX10-/IRF8+), 274 DoubleNeg (NeuN-/SOX10-), and 15 TripleNeg (NeuN-/SOX10-/IRF8-) nuclei samples were processed.

### DNAm data preprocessing and quality control

DNAm data was loaded into R (version 3.6.3) from IDAT files using the package bigmelon [28]. These data were processed through a bespoke quality control pipeline developed for cell-specific DNAm data. The pipeline is structured into three stages:

Stage 1—confirming the quality of the DNAm data.

Stage 2—confirming the correct individual.

Stage 3—confirming the correctly labelled cell type.

The first two stages are common to most existing pre-processing pipelines. The final stage of the pipeline confirms that FANS isolation was successful and fractions representing distinct cell types were obtained. It leverages the fact that as cell-type identity is the primary source of variation in DNAm profiles [8, 10, 29], the major principal components (PCs) should cluster the samples by cell type. Using the first two PCs, the distance (in SD units) between each sample and the mean profile of its labelled cell type is used to identify instances where either the FANs isolation was not successful (and heterogeneous samples were collected) or where samples have potentially been mislabelled. Full details on the steps within each stage can be found in Supplementary Text S1.

After stringent quality control, 751 samples were retained: 218 Total, 164 DoubleNeg, 182 NeuNPos, 168 Sox10Pos, 12 IRF8Pos, and 7 TripleNeg samples.

### Comparison of normalization strategies

This analysis was limited to cell types that had more than 100 samples (NeuNPos, Sox10Pos, and DoubleNeg), which were normalized using the *dasen*() function in the wateRmelon R package [30] in two ways:

(i) All samples from all cell types were normalized as a single dataset; and
(ii) Normalization was performed for each cell type separately.

These strategies were compared using three quantitative metrics proposed in the original wateRmelon manuscript [30] where, lower scores indicate a higher signal-to-noise ratio and a more effective normalization strategy (further details in Supplementary Text S2). In addition, we quantified the magnitude of transformation per sample, using the *qual*() function from the bigmelon package [28].

### Power calculations

We conducted power calculations to assess the impact of isolating purified nuclei populations on statistical power, using the function *pwr.t.test()* from the R package pwr [31]. We consider the scenario with a binary outcome (i.e. case control study), using a two-sample *t*-test to compare the means of the two groups. We profiled the effect of varying sample size or mean difference between groups on statistical power for each cell type separately, using cell-specific SDs for each site. The significance level was set to an experiment-wide threshold of *P* < 9 × 10^−8^ [32].

To get an overall power estimate for a specific scenario for study with 846 232 autosomal sites, we calculated the cumulative percentage of sites that had a minimum level of statistical power. To reduce the computational burden of performing >800 000 calculations per cell type, we leveraged the fact that many sites have similar SDs. Sites were assigned to 500 bins of equal size (i.e. same number of sites per bin) and containing sites with similar SDs. The mean of the SDs across sites within each bin is used to calculate Cohen’s d for the power calculation and the resulting power statistic is applied to all sites in that bin. When analyzing how the mean difference affected statistical power, we fixed the sample size at either 100 or 200 per group and evaluated a range of increasing mean differences (0.001, 0.002, 0.003, …, 0.1). When analyzing how sample size affected statistical power, we fixed the mean difference between groups (0.02 or 0.05) and evaluated 100 equally spaced sample sizes ranging from 0 up to the sample size needed to detect a minimum of 85% of sites with at least 80% power, rounded to the nearest 100. Note that in these results, we report the mean difference for DNAm measured as percentage points (i.e. bounded between 0 and 100) rather than as a proportion (i.e. bounded between 0 and 1).

### Simulation study to assess statistical frameworks for cell-specific EWAS

To assess the different analytical frameworks, we implemented two simulation scenarios. First, we generated 100 null association studies by randomly assigning samples as either ‘cases’ or ‘controls’. Second, taking each simulated null association study, we introduced a fixed number of differentially methylated positions (DMPs) at a random subset of sites, predetermined to be either common (i.e. affect all cell types) or specific to a single cell type. Further details can be found in Supplementary Text S3.

For each simulation, we compared four regression frameworks:

(i) Linear regression model for each cell type separately (within cell-type linear regression `ctLR'). As there is only one sample per individual per cell type, the observations can be considered to be independent.
(ii) Linear regression model using all samples from all cell types (‘allLR’). As there are multiple samples per individual, this approach is potentially biased.
(iii) Mixed effects linear regression model using all samples from all cell types (mixed effects regression `MER') where a random intercept accounts for multiple samples per individual.
(iv) Clustered robust regression (CRR) model using all samples from all cell types (‘CRR’), where samples from each individual are modelled as a cluster to account for the lack of independence.

All models included age, sex, and brain bank as covariates. Further details can be found in Supplementary Text S4. For each simulation and regression model, we recorded the number of significant associations at epigenome-wide significance (9×10^−8^) [32] as well as three discovery thresholds (1×10^−7^, 1×10^−6^, and 1×10^−5^), classifying these as either false positives or true positives.

## Results

### Novel quality control pipeline for processing cell-specific DNA methylation data

**Figure 1:**
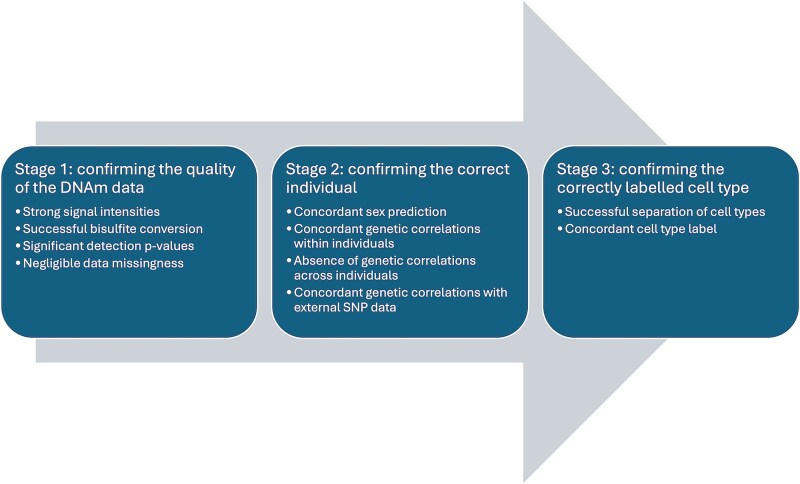
Overview of stages in quality control pipeline for cell-specific epigenetic data.

To ensure confidence and reliability in cell-sorted DNAm data, we developed a three-stage custom pipeline (Fig. 1; see Supplementary Text S1). Stage 1 confirms that the assay has generated high quality DNAm data. Stage 2 confirms that the sample matches their labelled individual by checking concordance with sex and genotype information. Stage 3 is tailored to the cell-sorted dimension of the dataset to confirm that FANS isolation was successful. For this, two novel metrics based on PCs were developed that quantify how closely each sample matches the average profile for its labelled cell type, using PCs. First, an individual level ‘isolation efficiency score’ was defined to verify that distinct fractions of nuclei were isolated for each FANs sort. Visual inspection of this score applied to an exemplar dataset determined a threshold of 5 was appropriate to identify individuals where sorting was unsuccessful and heterogeneous samples were collected (Supplementary Fig. S2). Second, we calculated the distance between each sample and its labelled cell type, retaining only those that were within two SDs of the cell type mean for the first two PCs.

### Increased sensitivity for detecting differentially methylated positions following normalization within cell types

Normalization is used to minimize experimental technical variation by transforming samples to a common standard, making them quantitatively comparable and maximizing the statistical sensitivity. While studies report little impact of normalization strategy on DMP detection in EWAS of single sample types [33, 34], dramatic differences in DNAm profiles between cell types may lead to unpredictable behaviour if these data are forced to become more similar to each other. We compared the effect of normalizing across all samples from all cell types together to normalizing separately within each cell type using an established framework [30]. Across three metrics, where lower scores indicate better performance, normalizing the data separately for each cell type was the optimal approach (Table 1). Of note, NeuNPos samples are subject to a much larger transformation (mean across sites = 0.039, SD = 0.016), when normalized with the other cell types, compared to when they are normalized only with NeuNPos samples (mean = 0.027; SD = 0.011; Supplementary Fig. S3). In contrast, DoubleNeg and Sox10Pos samples were associated with similar levels of transformation when normalized separately by cell type (DoubleNeg mean = 0.033; SD = 0.013; Sox10Pos mean = 0.027; SD = 0.012) compared to when normalized altogether (DoubleNeg mean = 0.032; SD = 0.014; Sox10Pos mean = 0.026; SD = 0.011). This is consistent with the knowledge that the primary axis of variation in sorted PFC samples captures differences between NeuN positive and NeuN negative samples [29] meaning these samples require the largest manipulation to make them comparable.

**Table 1:** Summary metrics to compare normalization strategies for cell-sorted DNAm data, where each column presents the results of a different metric proposed by Pidsley *et al*. to quantitative compare different normalization algorithms.

Normalization strategy	Metric		
	DMRSE	GCOSE	Seabird
	Imprinted regions	SNP probes	X-chromosome inactivation
Raw unnormalized data	1.578E-03	5.724E-05	0.053
Normalize all samples from all cell types together	1.600E-03	5.716E-05	0.037
Normalize each cell type separately	1.243E-03	5.107E-05	0.036

### Isolating homogeneous populations of cells reduces the number of samples required to reliably detect differentially methylated positions

It is often assumed that cell sorting is impractical for EWAS due to the large sample sizes required but extrapolating from power calculations derived from heterogeneous bulk tissues may be misleading. To test the hypothesis that purified cell populations have lower variance translating to increased power to detect cell-type-specific differences, we quantified probe level variation by cell type. Comparing the distribution of SDs across autosomal sites by cell type (Supplementary Fig. S4), lower variation across samples was observed as expected for the NeuNPos (mean = 0.036; SD = 0.023) and Sox10Pos (mean = 0.038; SD = 0.024) fractions relative to the Total samples (mean = 0.046; SD = 0.026). Total contains nuclei from all fractions and therefore can be considered a proxy for bulk PFC tissue. Of note the DoubleNeg (mean = 0.057; SD = 0.033) and IRF8Pos (mean = 0.055; SD = 0.036) fractions exhibit on average higher levels of variation with the TripleNeg (mean = 0.044; SD = 0.029) fraction showing similar levels of variation to Total.

**Figure 2:**
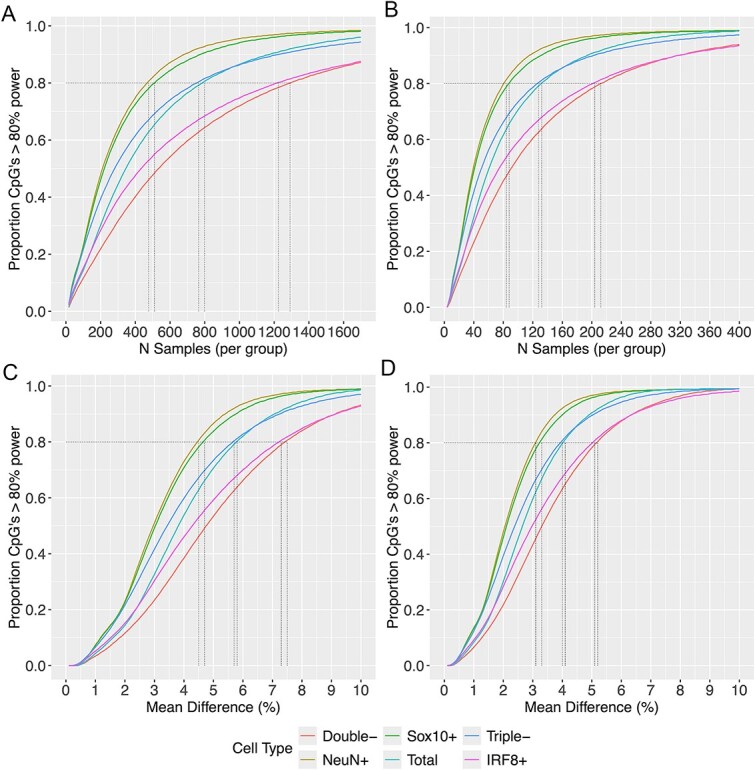
Cell-specific epigenetic association analyses increase statistical power over bulk tissue studies, as illustrated by power curves for purified brain cell types and bulk brain tissue across different experimental parameters, with Panels A and B showing power (y-axis) versus sample size for mean group differences of 5% and 2%, respectively, and Panels C and D showing power versus mean difference for fixed sample sizes of 100 and 200 cases versus controls, respectively, where each line represents a brain cell type or bulk tissue.

The reduced variation naturally has a positive effect on power and sample size requirements (Fig. 2). For example, to detect at mean difference of 5% points between groups with power of 80% at 80% of probes on the EPIC array, you need 84 samples per group for NeuNPos or 88 samples per group for Sox10Pos (Supplementary Table S1). In contrast, 132 Total, 128 TripleNeg, 204 IRF8Pos, or 212 DoubleNeg samples per group are required. Instead to detect a 2% difference, you need 476 samples per group for NeuNPos compared to 799 samples for Total or 1224 samples per group for IRF8Pos. Alternatively keeping the sample size constant at 100 samples per group, 80% power is obtained at 80% of sites to detect a mean difference of 4.5% for NeuNPos, 4.7% for Sox10Pos, 5.7% for TripleNeg, 5.8% for Total, 7.3% for IRF8Pos, and 7.5% for DoubleNeg between groups. This decreases to a mean difference of 3.1% for NeuNPos, 3.3% for Sox10Pos, 4.0% for TripleNeg, 4.1% for Total, 5.1% for IRF8Pos, and 5.2% for DoubleNeg between groups if the sample size is increased to 200 samples per group.

### Cell-specific EWAS are prone to false positives if within individual study design is not adequately controlled for

Cell-specific EWAS require an analytical framework that can account for multiple samples per individual and model potentially different magnitudes of disease associated variation across cell types. To evaluate the impact of different regression models on statistical sensitivity, we designed a simulation framework to compare four approaches. The first approach (‘ctLR’) is linear regression within each cell type, where there is no correlation between samples to control for. It is a computationally cheap analysis per DNAm site but involves fitting one model per cell type and is unable to determine the cell-specificity of the effect. The second approach (‘allLR’) uses all the data available from all cell types in a linear regression model. This method violates the assumption of independent observations but by aggregating the data together could increase power to detect DMPs shared across cell types. The third approach (‘MER’) uses a mixed effects regression model to control for the structure within the data. The final approach (‘CRR’) uses a clustered robust regression model, an alternative approach that can account for related samples. The allLR, MER, and CRR methods all include both a main effect and interaction terms to capture DMPs with different impacts across cell types.

**Figure 3:**
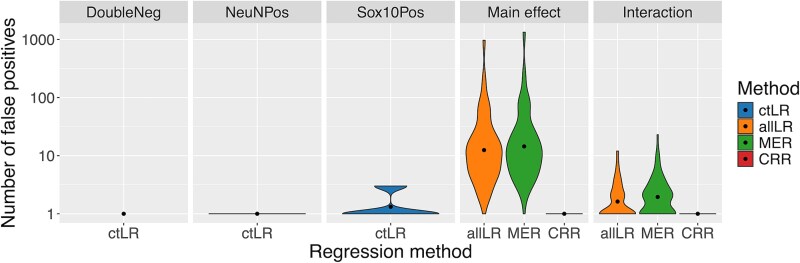
Choice of regression method is critical to minimize false positives in cell-specific association analyses, as shown by violin plots (y-axis: number of false positive DMPs on a log scale, *P* < 9 × 10^−8^) from 100 simulated null EWAS, where each violin represents a different statistical analysis, colored by regression method—ctLR (within cell-type linear regression), allLR (linear regression across all cell types), MER (mixed effects regression), and CRR (clustered robust regression).

When deciding upon an efficient method, there are two desirable properties: minimizing the false positive rate and maximizing the true positive rate. To quantify the false positive rate for each approach, we simulated 100 null EWAS (see Supplementary Text S2). For regression models that include all data from all samples (allLR, MER, and CRR), depending on which cell type(s) a DMP affects, both or either of the main effect and interaction term may be significant, so both terms must be considered when assessing these methods. We observed that the distribution of log *P*-values for both the allLR and MER models is wider with longer tails indicative of smaller mean *P*-values (Supplementary Fig. S5). Applying the standard threshold for epigenome-wide significance (9×10^−8^ [32]), we observed that the ctLR and CRR are well calibrated with all models identifying a mean of <0.1 false positive DMPs (Fig. 3). In contrast, both the allLR and MER models had increased rates. These were dramatically inflated for the main effect term (allLR mean = 42 (SD = 136), MER mean = 53 [SD = 185]) and subtly higher also for the interaction term (allLR mean = 1 (SD = 2); MER mean = 2 (SD = 3)). This highlights that the choice of analysis method is critical and has dramatic effects on the robustness of the results. One mechanism to counteract this is to derive a significance threshold calibrated specifically for each method. Calculating the mean 5% family-wise error rate for each method across the null simulations (Supplementary Fig. S6), the significance thresholds for the ctLR and CRR models were comparable to the standard threshold (range: *P* = 9.7×10^−8^–2.8×10^−7^), whereas the thresholds for the allLR and MER models needed to be 2–7 orders of magnitude smaller (range: *P* = 9.7×10^−16^–5.3 × 10^−9^).

**Figure 4:**
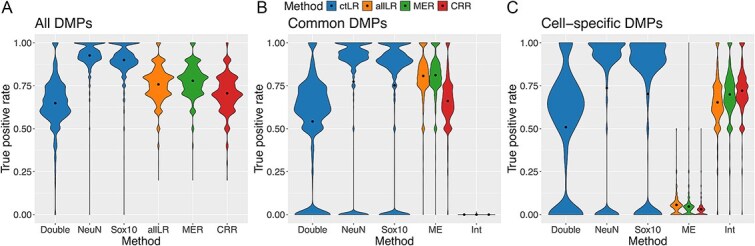
Violin plots compare true positive rates (*P* < 9 × 10^−8^) across cell-specific regression methods using results from 1,800 simulated EWAS in which 10, 100, or 1,000 differentially methylated positions (DMPs) with a 5% mean group difference were introduced, with varying proportions (0–1) of DMPs designated as cell-specific versus common across all cell types, and true positive rates are shown for (A) all DMPs, (B) cell-specific DMPs, and (C) common DMPs, with each violin representing a regression method—ctLR (within cell-type linear regression), allLR (linear regression across all cell types), MER (mixed effects regression), and CRR (clustered robust regression).

To assess each method’s accuracy at detecting true positive associations, we implemented a second simulation scenario where a fixed number of DMPs were introduced with effects that were either common to all three cell types or specific to just one. Considering DMPs with a mean difference of 5% between cases and controls, the ctLR in NeuNPos samples had the highest true positive rate (mean = 0.76, SD = 0.35; Fig. 4), slightly higher than the MER (mean = 0.74, SD = 0.09), ctLR in Sox10Pos samples (mean = 0.73, SD = 0.34), and allLR (mean = 0.72, SD = 0.09). There was then a drop in performance to the CRR method (mean = 0.66, SD = 0.10) followed by another drop in performance to the ctLR in DoubleNeg samples (mean = 0.49, SD = 0.26). The disparity in performance with the ctLR method for different cell types fits with the results of the power calculations, highlighting how the heterogeneity of DoubleNeg samples impacts the ability to detect genuine associations.

Generally, performance did not depend on whether a DMP was common to all cell types or specific to just one, although it is notable that the CRR model had a higher true positive rate for detecting cell-specific DMPs (mean = 0.68, SD = 0.13) than either the MER (mean = 0.65, SD = 0.14), or allLR (mean = 0.60, SD = 0.15). Regardless, these methods all performed slightly worse than the ctLR in either NeuNPos (mean = 0.72, SD = 0.36) or Sox10Pos (mean = 0.68, SD = 0.38) samples for these DMPs. The pattern of results was maintained when the mean difference between cases and controls was reduced to 2%, albeit with reduced true positive rates (Supplementary Fig. S7; range of mean true positive rates across all DMPs = 0.12–0.27). There was no meaningful effect of the number of DMPs on the performance of the regression method (Supplementary Fig. S8). Interestingly, as the proportion of cell-type-specific DMPs increased the true positive rate for the allLR and MER methods decreased and increased for the CRR method.

When EWAS fail to identify DMPs at the standard epigenome-wide significance threshold this is commonly attributed to a lack of statistical power. ‘Discovery thresholds’ are often used instead to uncover potential associations. However, since the validity of this approach is untested, we used our simulations to assess the potential benefits and identify a suitable discovery threshold. First, considering the number of false positives at a range of discovery thresholds (1×10^−7^, 1×10^−6^, and 1×10^−5^), the methods that had minimal false positives at epigenome-wide significance, ctLR and CRR, maintained a mean of less than 1 false positive up to a threshold of <10^−6^. This increased to between 4 and 10 at the most permissive *P*-value threshold of <10^−5^ (Supplementary Table S2). Meanwhile, the methods that already had elevated mean false positive counts continued to accumulate additional false positives, increasing from 43 to 656 for allLR and from 55 to 785 for MER at the most relaxed threshold (*P*-value <10^−5^). Secondly, considering the true positive rate, this increased as the significance threshold is relaxed for all methods, albeit to different degrees (Supplementary Table S3). There are smaller gains for the methods that were already associated with high rates, for example, ctLR in NeuNPos samples increases from 0.91 (SD = 0.08) at 9×10^−8^ to 0.95 (SD = 0.06) at 10^−5^ with bigger gains for methods that had lower rates at the most stringent threshold, e.g., CRR increases from 0.66 (SD = 0.10) at 9×10^−8^ to 0.78 (SD = 0.09) at 10^−5^.

## Discussion

Determining the cell types affected by differences in DNAm is the next critical step in enhancing our understanding of the role of epigenetics in health and disease. As cell-specific EWAS gain traction, now is the critical time to establish a framework that promotes statistically robust analyses facilitating the generation of meaningful and accurate biological insights. We present the first quantitative assessment of study design, quality control, and statistical analysis for cell-specific DNAm association studies. Using an exemplar dataset with five purified populations of nuclei obtained by FANS from post-mortem cortical tissue, we use both empirical power calculations and simulations to determine a statistically robust approach that identifies and characterizes the cell-specificity of positions in the genome associated with an outcome.

Balancing the need to minimize the false positive rate and maximize the true positive rate, we propose the following analytical strategy for EWAS of multiple cell types. First, we recommend performing a within cell type association analysis using linear regression. From each regression model (one per cell type), significant DMPs can be identified with low risk of false positive associations, subject to appropriate experimental design and adjustment for confounders. The caveat with this approach is that it cannot determine whether the identified DMP is cell type-specific or affects multiple cell types. Cross-referencing DMP lists across cell types may reveal overlaps, but a lack of overlap does not confirm absence of effect, as it could result from sampling variation or limited statistical power. Therefore, a second stage of the analysis is required to confirm whether the effect is common to all cell types by testing for differences or heterogeneity across cell types. We propose that a mixed effects model is used in this scenario with an interaction term and random effect to control for multiple samples per individual. While this method was associated with a high number of false positives, we would discourage using it to discover DMPs but instead to characterize the cell-type specificity of the effect. We additionally investigated the impact of using a ‘discovery threshold’ but this offered minimal benefit when identifying additional true positives. If required, we recommend not exceeding 10^−6^, as this kept the number of false positives below 1.

Our motivation is informed by challenges observed in single-cell transcriptomics, where multiple replicates per sample (on a substantially greater scale) and the identification of cell-specific differences pose significant analytic challenges. Analyses of both empirical and synthetic datasets in that domain have shown that some proposed methods, including as we did mixed effects models, fail to account adequately for the variability between replicates and consequently are associated with inflated counts of false positives [35]. While single-cell sequencing is a complementary strategy for identifying cell-specific effects, it is fundamentally a different approach and currently lacks a robust way for quantifying DNAm. Profiling a pool of molecules from the same cell type is instead, more accurate and cost effective than profiling a single molecule. By positively selecting specific populations of nuclei, we ensure that sufficient material is collected for efficient quantification. Our experimental approach is therefore not only technically more robust but also more scalable and economical, making it well-suited for large cohort epigenetic epidemiology studies.

One of our key take home messages is that isolating populations of cells may not need to be performed in as many samples as would be needed for a bulk tissue EWAS. Our power calculations found that for some purified populations, there were substantial gains in statistical power for detecting DMPs. Consequently, in studies with limited samples (e.g. post-mortem brain tissue), cell-sorting could be a valuable strategy to boost statistical power and maximize the impact of a limited resource. Within specific cell types, it is anticipated that effect sizes will be greater, enhancing power further. However, for some cell-sorted fractions, such as IRF8Pos fractions (microglia enriched) and TripleNeg fractions (astrocyte enriched), there was an increase in variation and therefore a decrease in power. This suggests that within these fractions, further isolation may be required to not only increase the probability of finding genuine associations, but also to clarify which cell types show increased variation in DNAm.

The findings we present should be mindful of the following limitations. First, we only considered a case-control study design, the most commonly used study design for disease associated epigenetic differences. As the regression methods we used are easily adaptable for a broad range of phenotypes, including both continuous and categorical outcomes we are confident that our results would also hold for these other EWAS scenarios, which will also have to account for the structure in the data. Second, while our data only consists of neural purified cell populations, we believe the overarching conclusions of our study are generalizable to cell types from other tissues. For those interested in DNAm EWAS of brain cell types, we have developed an R package, CellPower, for the community to perform power calculations for brain cell-specific EWAS. Third, we only considered a limited number of parameter combinations for DMPs and sample size. We believe this is sufficient to address the questions we posed, but caution should be applied when extrapolating from the specific true positive and false positive rates we report to other EWAS scenarios. Fourth, we used a microarray to quantify DNAm with limited coverage of CpGs. There are other sequencing based technologies that can be used to profile DNAm more extensively across the genome that generate estimates with different statistical properties [36] and it may be that our results do not extend to these other technologies. All of these limitations could be addressed with follow up studies that apply our simulation framework to other study designs, or cell-specific data generated from different tissues or technologies to confirm that our findings translate beyond the scenario we explored.

## Conclusion

In conclusion, our analyses provide a valuable insight into the potential of cell-specific DNAm association analyses and provide a benchmark against which future studies can be evaluated in terms of their study design, quality control pipelines and statistical analysis.

**Key Points**

- We propose an analytical framework for cell-specific DNAm data that incorporates bespoke quality control metrics and not only performs an association analysis to identify differentially methylation positions but that also characterizes whether it affects multiple cell types or is specific to a particular cell type.
- Consideration must be given to the most appropriate analytical model for these data, as even methods that theoretically adjust for correlations between samples, may be associated with inflated rates of false positives.
- Isolating purified cell types can lead to increases in statistical power to identify differentially methylated positions and therefore this might be an effective way of maximize the value of a limited resource such as post-mortem brain tissue samples.

## Supplementary Material

Supplementary_Figures_bbaf427

SupplementaryTables_bbaf427

SupplementaryText_bbaf427

## Data Availability

Raw and processed DNAm data are available from GEO under accession number GSE279509.

## Code availability

All code for the results presented in this manuscript are available at https://github.com/ejh243/BrainFANS. The bespoke quality control pipeline we describe can be found at https://github.com/ejh243/BrainFANS/tree/master/array/DNAm/preprocessing, codes for the analyses can be found at https://github.com/ejh243/BrainFANS/tree/master/array/DNAm/analysis/methodsDevelop-ment. We have also made our data and method for cell-specific EWAS power calculations available as a standalone R package which is available at https://github.com/ew367/CellPower/tree/main.
